#  Mitochondrial Toxicity of Depleted Uranium: Protection by Beta-Glucan 

**Published:** 2013

**Authors:** Fatemeh Shaki, Jalal Pourahmad

**Affiliations:** a *Faculty of Pharmacy, Shahid Beheshti University of Medical Sciences, Tehran, Iran.*; b * Faculty of Pharmacy, Manzandaran University of Medical Sciences, Sari, Iran.*; c *Students Research Committee, School of Pharmacy Shahid Beheshti University of Medical Sciences, Tehran, Iran.*; d *Pharmaceutical Sciences Research Center, Shahid Beheshti University of Medical Sciences, Tehran, Iran. *

**Keywords:** Depleted uranium, Beta-glucan, Mitochondria, Nephrotoxicity, Protection, Antioxidant

## Abstract

Considerable evidence suggests that mitochondrial dysfunction contributes to the toxicity of uranyl acetate (UA), a soluble salt of depleted uranium (DU). We examined the ability of the two antioxidants, beta-glucan and butylated hydroxyl toluene (BHT), to prevent UA-induced mitochondrial dysfunction using rat-isolated kidney mitochondria. Beta-glucan (150 nM) and BHT (20 nM) attenuated UA-induced mitochondrial reactive oxygen species (ROS) formation, lipid peroxidation and glutathione oxidation. Beta-glucan and BHT also prevented the loss of mitochondrial membrane potential (MMP) and mitochondrial swelling following the UA treatment in isolated mitochondria. Our results show that beta-glucan and BHT prevented UA-induced mitochondrial outer membrane damage as well as release of cytochrome c from mitochondria. UA also decreased the ATP production in isolated mitochondria significantly inhibited with beta-glucan and BHT pre-treatment. Our results showed that beta-glucan may be mitochondria-targeted antioxidant and suggested this compound as a possible drug candidate for prophylaxis and treatment against DU-induced nephrotoxicity.

## Introduction

Depleted uranium (U) is a by-product of the uranium enrichment that has removed most of its radioactive isotopes U_235_ and U_234_ (1). This depleted form of uranium has about 60% of the radioactivity of the natural U and its density, availability, and relatively low cost make it attractive for military purposes, specifically in anti-armor weapons and projectiles (2). This military use has resulted in exposures to DU through respiration, ingestion and wound contamination. DU, like other heavy metals, is nephrotoxic and can accumulate in the kidney tissue and injured proximal tubular epithelial cells (3). The most important toxic mechanism suggested for DU toxicity is the involvement of oxidative stress and reactive oxygen species (ROS) (4-6). Previous studies showed that the oral uranyl acetate (UA) administration increases the TBARS (thiobarbituric acid reactive substances) in kidney and testis (7). Other studies have revealed that the chronic uranyl nitrate ingestion results in an increase in the level of free radicals (8) and lipid peroxidation in CNS (9) and rapid oxidation of glutathione, ROS formation, lipid peroxidation and also decreases the mitochondrial membrane potential (MMP) in isolated rat hepatocytes (5).

Mitochondria are the major source of ROS in most mammalian cell types (10) and also key organelles in the development of cellular oxidative damage. Previous studies demonstrated the significant MMP collapse (5, 11) and mitochondrial swelling (12) after DU exposure in different cell lines. Therefore, mitochondrial dysfunction and oxidative damage may be responsible for the pathological consequences of DU exposure in living organism.

There are a lot of antioxidants introduced for their preventive ability against oxidative stress damage. We have focused this investigation on the role of beta-glucan and BHT as antioxidants and protective agents against mitochondrial oxidative damage.

Glucans or polymers of D-glucose linked by b-(1-->3) and b-(1-->6) glycosidic linkages are cell wall polysaccharides in many microorganisms, fungi and algae, and also well-known biological response modifiers. Beta-glucan showed beneficial effects on the immune system and lacks any toxic or adverse effects (13-16). These compounds exhibited antitumor effects and prevention of carcinogenesis increase in the host resistance to infections (17). Recently, it was found that beta-glucan and its derivatives are antioxidant with the scavenging ability (14-16, 18). Beta-glucan, due to its polymeric structure, can trap free radicals and has antioxidant and free radical scavenger properties. The antioxidant capacity of the molecule is the most important mechanism proposed for the protective effects of beta-glucan (19).

Butylated hydroxyl toluene (BHT) is a known antioxidant commonly used as synthetic antioxidants in foods (20). Previous studies showed the ability of BHT in protection mitochondria against oxidative damage. BHT significantly inhibited oxidative damage, MMP collapse and the release of cytochrome c from isolated mitochondria after exposure to various oxidative agents (21-24).

However, there are no reports on the protective effect of beta-glucan against mitochondrial oxidative stress and also DU-nephrotoxicity*. *Therefore, in the present study we investigated the protective role of beta-glucan in DU-induced mitochondrial dysfunction using isolated kidney mitochondria and BHT used as positive control.

## Experimental

*Materials*

Uranyl acetate (U238 = 99.74%, U235 = 0.26%, U234 = 0.001%), with 1.459E4 Bq/g specific activity based on manufacturer data), butylated hydroxyl toluene (BHT), beta-glucan, 4-2-hydroxyethyl-1-piperazineethanesulfonic acid (HEPES), D-mannitol, thiobarbituric acid (TBA), MTT (3-[4,5-dimethylthiazol-2-yl]-2,5-diphenyltetrazolium bromide), dithiobis-2-nitrobenzoic acid (DTNB), reduced glutathione (GSH), 2’,7’-dichlorofluorescein diacetate (DCFH-DA), Malondialdehyde (MDA), Tris-HCl, sodium succinate, sulfuric acid, *n*-butanol, Tetramethoxypropane (TEP), KCl, Na_2_HPO_4_, MgCl_2_, MnCl_2_, potassium phosphate, Rhodamine 123 (Rh 123), Coomassie blue, Ethylene glycol-bis (2-aminoethylether)-N,N,N´,N´-tetraacetic acid (EGTA), ethylenediaminetetraacetic acid (EDTA) and bovine serum albumin (BSA) were purchased from Sigma Chemical Co. (St. Louis, MO, USA). All chemicals were of analytical, HPLC or the best pharmaceutical grades.

*Animals’ treatment*

Male Wistar rats (250-300 g) were housed in an air-conditioned room with controlled temperature of 25 ± 2°C and maintained on a 12:12 h light cycle with free access to food and water. All experimental procedures were conducted according to the ethical standards and protocols approved by the Animal Experimentation Committee of Shahid Beheshti University of Medical Sciences, Tehran, Iran. All efforts were made to minimize the number of animals and their suffering.

*Mitochondrial preparation*

Mitochondria were prepared from Wistar rat’s kidneys using differential centrifugation (25). Tissues were minced and homogenized with glass hand-held homogenizer. The nuclei and broken cell debris were sedimented through centrifuging at 1500×g for 10 min at 4ºC and the pellet was discarded. The supernatant was subjected to a further centrifugation at 10,000×g for 10 min and the superior layer was carefully discarded. The mitochondrial pellet was washed by gently suspending in the isolation medium and centrifuged again at 10,000×g for 10 min. Final mitochondrial pellets were suspended in Tris buffer containing (0.05 M Tris-HCl, 0.25 M sucrose, 20 Mm KCl, 2.0 mM MgCl_2_, and 1.0 mM Na_2_HPO_4_, pH of 7.4) at 4°C, except for the mitochondria used to assess ROS production, MMP and swelling, which were suspended in respiration buffer (0.32 mM sucrose,10 mM Tris, 20 mM Mops, 50 μM EGTA, 0.5 mM MgCl2, 0.1 mM KH_2_PO_4_ and 5 mM sodium succinate ), MMP assay buffer (220 mM sucrose, 68 mM D-mannitol, 10 mMKCl,5 mM KH_2_PO_4_, 2 mM MgCl_2_, 50 μM EGTA, 5 mM sodium succinate, 10 mM HEPES, 2 μM Rotenone) and swelling buffer (70 mM sucrose, 230 mM mannitol, 3 mM HEPES, 2 mM tris-phosphate, 5 mM succinate and 1 μM of rotenone). Protein concentrations were determined through the Coomassie blue protein-binding method as explained by Bradford, 1976 (26). The isolation of mitochondria was confirmed by the measurement of succinate dehydrogenase (27) .Mitochondria were prepared fresh for each experiment and used within 4 h of isolation and all steps were strictly operated on ice to guarantee the isolation of high-quality mitochondrial preparation.

Uranyl acetate (UA), a soluble form of DU, was used in our study due to our interest in environmental exposure. It also releases a more neutral anion compared to uranyl nitrate which is less soluble and more oxidizing (28). UA was dissolved in distilled water. The concentrations of UA (50, 100, 200 μmol/L) were chosen based on the previous study (5) and mitochondrial fractions were incubated in Tris buffer with different concentrations of UA for 1 h. In experiments where BHT and beta-glucan were employed, mitochondria were pre-incubated with the BHT and beta-glucan was added to the incubation mixtures for 5 min before the UA.

*Quantification of mitochondrial ROS level*

The mitochondrial ROS measurement was performed using the fluorescent probe DCFH-DA. Briefly, isolated kidney mitochondria were incubated with UA (0, 50, 100 and 200 μM) in respiration buffer containing (0.32 mM sucrose, 10 mM Tris, 20 mM Mops, 50 μM EGTA, 0.5 mM MgCl_2_, 0.1 mM KH_2_PO_4_ and 5 mM sodium succinate ) (29). Following the UA incubation, a sample was taken and DCFH-DA was added (final concentration, 10 μM) to mitochondria and then incubated for 10 min and the fluorescence intensity of DCF was measured using Shimadzu RF-5000U fluorescence spectrophotometer at an excitation wavelength of 488 nm and emission wavelength of 527 nm.

*Measurement of GSH content*

GSH content was determined using DTNB as the indicator and spectrophotometer method for the isolated mitochondria. The mitochondrial fractions (0.5 mg protein/mL) were incubated with various concentrations of uranyl acetate for 1 h at 30ºC and then 0.1 mL of mitochondrial fractions was added into 0.1 mol/L of phosphate buffer and 0.04% DTNB in a total volume of 3.0 mL (pH = 7.4). The developed yellow color was read at 412 nm on a spectrophotometer (UV-1601 PC, Shimadzu, Japan). GSH content was expressed as μg/mg protein (30).

*Determination of the MMP*

Mitochondrial uptake of the cationic fluorescent dye, rhodamine 123, has been used for the estimation of mitochondrial membrane potential. The mitochondrial fractions (0.5 mg protein/mL) were incubated with various concentrations of uranyl acetate and then 10 μM of rhodamine 123 was added to mitochondrial solution in MMP assay buffer (220 mM sucrose, 68 mM D-mannitol, 10 m MKCl,5 mM KH_2_PO_4_, 2 mM MgCl_2_, 50 μM EGTA, 5 mM sodium succinate, 10 mM HEPES, 2 μM Rotenone). The fluorescence was monitored using Shimadzu RF-5000U fluorescence spectrophotometer at the excitation and emission wavelength of 490 nm and 535 nm, respectively (31).

*Determination of mitochondrial swelling*

Analysis of mitochondrial swelling after the isolated mitochondria (0.5 mg protein/mL) was estimated through changes in light scattering as monitored spectrophotometrically at 540 nm (30°C) as described (32). Briefly, isolated mitochondria were suspended in swelling buffer (70 mM sucrose, 230 mM mannitol, 3 mM HEPES, 2 mM tris-phosphate, 5 mM succinate and 1 μM of rotenone) and incubated at 30°C with 50, 100 and 500 μM of uranyl acetate. The absorbance was measured at 549 nm at 10 min time intervals with an ELISA reader (Tecan, Rainbow Thermo, Austria). A decrease in absorbance indicates an increase in mitochondrial swelling. 

*Measurement of outer mitochondrial membrane damage*

Outer membrane integrity was evaluated using cytochrome c oxidase assay kit (Sigma, St. Louis, MO). The colorimetric assay was based on the observation that a decrease in absorbance of ferrocytochrome c at 550 nm was caused by its oxidation to ferricytochrome c by cytochrome c oxidase. 

Mitochondrial outer membrane integrity was assessed through measuring the cytochrome c oxidase activity of mitochondria in the presence or absence of the detergent, *n*-dodecyl *β-*D-maltoside. The mitochondrial outer membrane damage was assayed from the ratio between cytochrome c oxidase activity with and without detergent. 

*Assay of ATP and ATP/ADP ratio*

The ATP and ATP/ADP ratio level were measured by luciferase enzyme as described by Tafreshi *et al. *2007 (33). Bioluminescence intensity was measured using Sirius tube luminometer (Berthold Detection System, Germany). 

*Cytochrome-c release assay*

The concentration of cytochrome c was determined through using the Quantikine Rat/ Mouse Cytochrome c Immunoassay kit provided by R and D Systems, Inc. (Minneapolis, Minn.). Briefly, a monoclonal antibody specific for rat/mouse cytochrome c was pre-coated onto the microplate. Seventy-five μL of conjugate (containing monoclonal antibody specific for cytochrome c conjugated to horseradish peroxidase) and 50 μL of standard and positive control were added to each well of the microplate. One microgram of protein from each supernatant fraction was added to the sample wells. All of the standards, controls and samples were added to two wells of the microplate. After 2 h of incubation, the substrate solution (100 μL) was added to each well and incubated for 30 min. After 100 μL of the stop solution was added to each well; the optical density of each well was determined through the aforementioned microplate spectrophotometer set to 450 nm. 

*Statistical Analysis*

Results are presented as mean ± SD. All statistical analyses were performed using the SPSS software, version 17. Assays were performed in triplicate and the mean was used for statistical analysis. Statistical significance was determined using the one-way ANOVA test, followed by the post-hoc Tukey test. Statistical significance was set at p < 0.05. 

## Results

**Figure 1:**
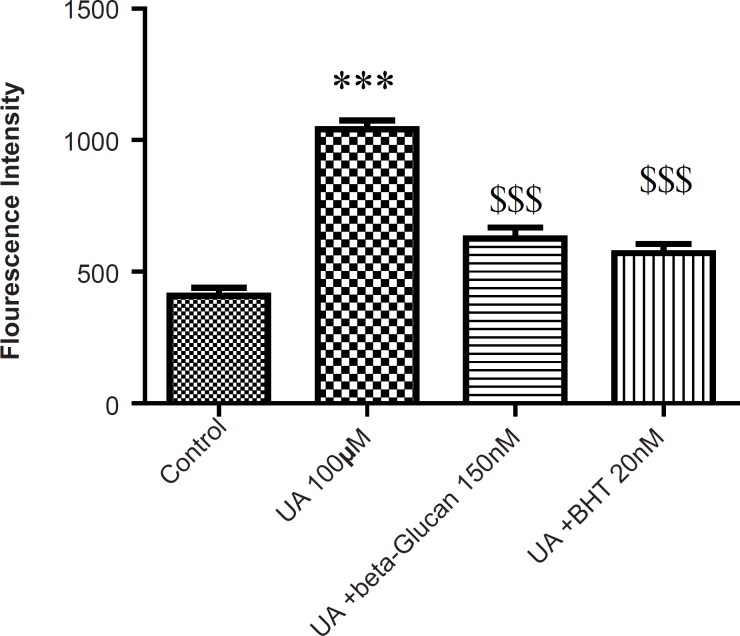
Preventing uranyl acetate (UA)-induced ROS formation by beta-glucan and BHT. ROS formation was measured fluorometrically using DCF-DA as described in Experimental. Values are presented as mean ± SD (n = 3). *: Significant difference in comparison with control mitochondria (p < 0.05). $: Significant difference in comparison with UA-treated mitochondria (p < 0.05).

As shown in Figure 1, following the UA exposure, ROS generation was significantly increased in isolated kidney mitochondria, while ROS formation was significantly (p < 0.05) inhibited by pre-treatments beta-glucan (150 nM) and BHT (20 nM). 

**Figure 2:**
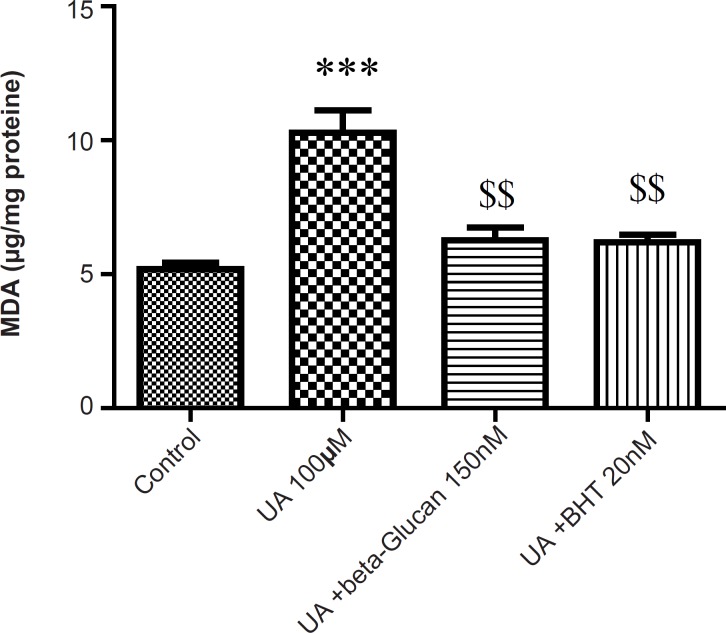
Preventing uranyl acetate (UA)-induced lipid peroxidation by beta-glucan and BHT. Lipid peroxidation was measured as thiobarbituric acid reactive substances. The assay was described in Experimental. Values are presented as mean ± SD (n = 3). *: Significant difference in comparison with control mitochondria (p < 0.05). $: Significant difference in comparison with UA-treated mitochondria (p < 0.05

Malondialdehyde (MDA) is the final product of lipid peroxidation (LPO) that is often used as an indicator of oxidative damage. As shown in Figure 2, in isolated kidney mitochondria, MDA was markedly increased following the UA exposure and both beta-glucan (150 nM) and BHT (20 nM) treatments significantly (p < 0.05) prevented UA-induced mitochondrial lipid peroxidation. 

**Figure 3:**
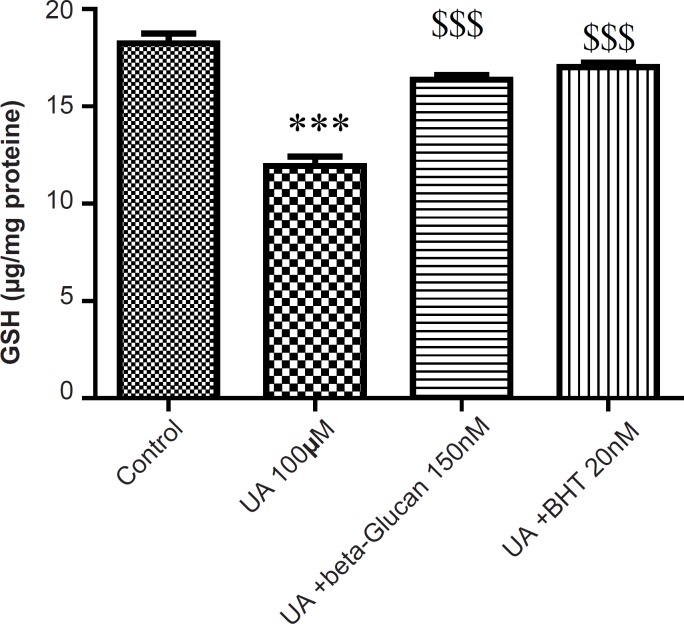
Preventing uranyl acetate (UA)-induced GSH oxidation by beta-glucan and BHT. GSH oxidation was measured as described in Experimental. Values are presented as mean ± SD (n = 3). *: Significant difference in comparison with control mitochondria (p < 0.05). $: Significant difference in comparison with UA-treated mitochondria (p < 0.05).

The mitochondrial GSH content was found to be decreased as a consequence of mitochondrial ROS formation in UA-treated mitochondria compared with the control group (p < 0.05). Again, pretreatment of both beta-glucan (150 nM) and BHT (20 n M) significantly (p < 0.05) inhibited the UA-induced mitochondrial GSH oxidation (Figure 3). 

**Figure 4:**
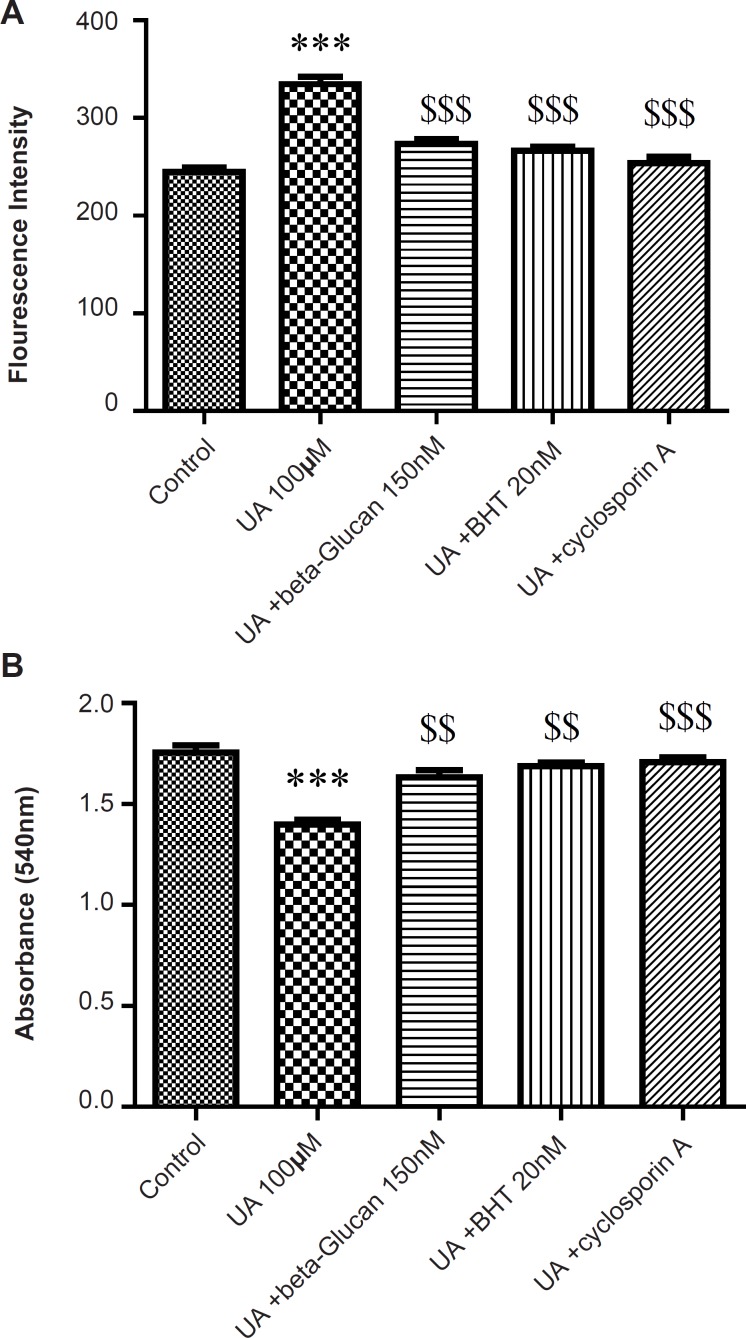
Preventing uranyl acetate (UA)-induced mitochondrial outer membrane damage by beta-glucan and BHT. Mitochondrial outer membrane damage was measured as described in *Experimental*. Values are presented as mean ± SD (n = 3). *: Significant difference in comparison with control mitochondria (p < 0.05). $: Significant difference in comparison with UA-treated mitochondria (p < 0.05).

The mitochondrial membrane damage was found to be significantly (p < 0.05) higher in the UA-treated isolated mitochondria compared with control mitochondria. Pre-treatment with beta-glucan (150 nM) and BHT (20 nM) prevented the UA-induced mitochondrial membrane damage (Figure 4). 

**Figure 5:**
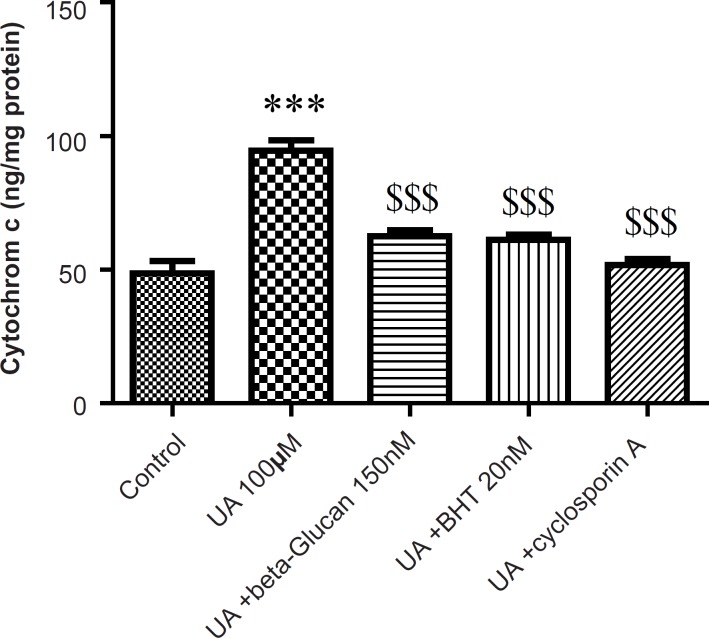
Preventing uranyl acetate (UA)-induced mitochondrial permeability transition by beta-glucan and BHT. A) MMP was measured by fluorogenic cationic probe rhodamine 123 and B) Mitochondrial swelling was measured through the determination of absorbance at 540 nm as described in Experimental. Values are presented as mean ± SD (n = 3).

MMP is an electrochemical potential that consists of a transmembrane electrical potential and a proton gradient. MMP collapse is an early sign of mitochondrial dysfunction (29). UA significantly induced mitochondrial membrane potential (MMP) collapse (p < 0.05) that was reversed by beta-glucan (150 nM) pretreatment (Figure 5). In addition, cyclosporine A markedly prevented UA-induced mitochondrial MMP collapse (p < 0.05) (Figure 5). 

The absorbance changes of mitochondrial suspensions are shown in Figure 5B due to MPT after the treatment with UA (100 μM). UA induced swelling (decrease of absorbance) in isolated kidney mitochondria. Pre-treatment of mitochondria with beta-glucan for 5 min decreased UA-induced swelling. Similarly, cyclosporine A (a MPT inhibitor) reversed the UA-induced swelling in isolated kidney mitochondria. The antioxidant, BHT, reversed MMP collapse by UA and also inhibited the mitochondrial swelling in the presence of UA (Figure 3) consistent with previous studies (21, 22). 

**Figure 6:**
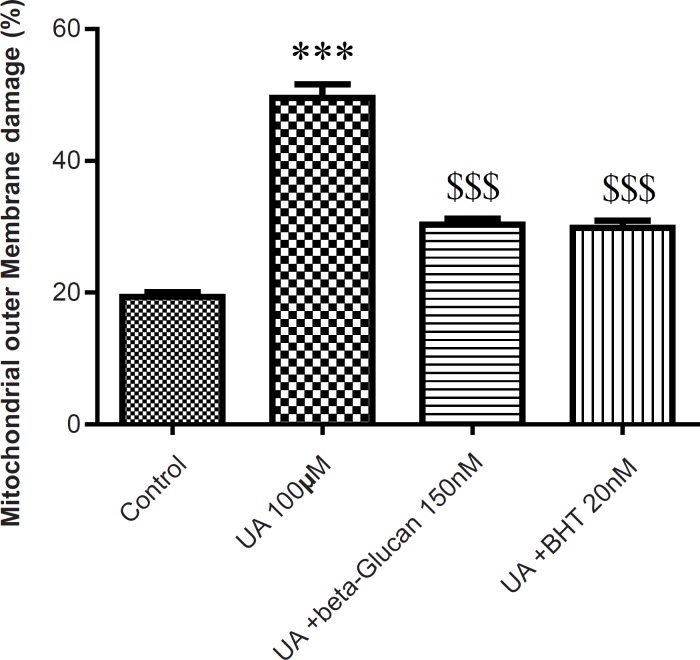
Preventing uranyl acetate (UA)-induced cytochrome c release by beta-glucan and BHT. Cytochrome c release was measured by ELISA kit as described in Experimental. Values are presented as mean ± SD (n = 3). *: Significant difference in comparison with control mitochondria (p < 0.05). $: Significant difference in comparison with UA-treated mitochondria (p < 0.05).

Cytochrome c release, the endpoint of mitochondrial toxicity, was significantly (p < 0.05) increased in the UA-treated mitochondria compared to control group (Figure 6). Release of cytochrome c from mitochondria was significantly (p < 0.05) inhibited with beta-glucan (150 nM) that showed similar effect with BHT (Figure 6). 

Opening of mitochondrial PT pores initiates onset of the mitochondrial permeability transition (MPT) which not only induces mitochondrial depolarization and mitochondrial swelling but also leads to uncoupling of oxidative phosphorylation. 

**Figure 7:**
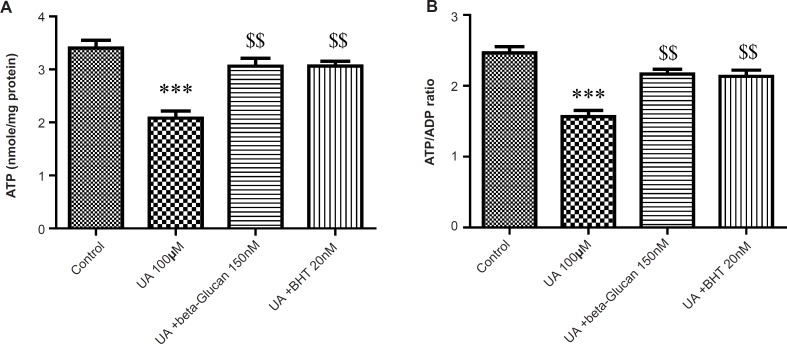
Preventing uranyl acetate (UA)-induced ATP depletion by beta-glucan and BHT. (A) ATP concentration and (B) ATP/ADP ratio were determined by *Luciferin/Luciferase *Enzyme System as described in Experimental. Values are presented as mean ± SD (n = 3). *: Significant difference in comparison with control mitochondria (p < 0.05). $: Significant difference in comparison with UA-treated mitochondria (p < 0.05).

As shown in Figure 7 A and B, mitochondrial ATP concentration and ATP/ADP ratio was significantly decreased by UA (p < 0.05). These effects were reversed by beta-glucan (150 nM) and BHT pretreatments (p < 0.05). 

## Discussion

Recently, antioxidants have been subjected to many studies that have connected their consumption to a reduction in the incidence of oxidative damage-related diseases. Therefore, much attention has been focused on the use of antioxidants, especially natural antioxidants, for the improvement of human health (34). 

The role of mitochondria in UA toxicity has been well established (4-6, 11, 35). Mitochondria are the main source for ROS production and also considered as targets of oxidative damage (36, 37). In fact, mitochondrial ROS could cause damage to mitochondrial structures and function, due to their very high reactivity. One possible explanation for UA-induced mitochondrial dysfunction is that the mitochondrial antioxidant system is not efficient at detoxifying relevant ROS. Several studies exhibited that the beta-glucan (14-16, 18) acts as an antioxidant with a protective effect against the lipid peroxidation in *e.g*., burn-induced oxidative skin damage and pressure ulcers in rats (13, 17, 38). In another study, beta-glucan significantly prevented the lipid peroxidation and cellular injury, reversed the altered antioxidant parameters and protected the skin against 2.45-GHzEMR-induced oxidative damage (17). Beta-glucan also showed antioxidant effects on the streptozotocin-induced diabetic rats. They significantly inhibited the increase in MDA, oxidative stress and reduction in total antioxidant status in brain and sciatic nerve (39). 

To study the protective effect of beta-glucan and BHT on mitochondrial function, we used the isolated rat kidney mitochondria to assess these compounds on UA-induced mitochondrial ROS formation and oxidative damage. 

Our results showed that UA-induced ROS production, lipid peroxidation and GSH oxidation in isolated kidney mitochondria were attenuated by beta-glucan and BHT (Figure 1-3). Our results were in accordance with several reports that beta-glucan could prevent oxidative stress in *in-vitro *models. Toklu *et al*. showed that both systemic and local administration of beta-glucan were effective against burn-induced oxidative damage in rat and significantly reversed the elevations in MDA levels and reduced GSH levels (19). Beta-glucan also significantly restored the reduced GSH levels and protected against nicotine-induced oxidative damage in rat (13). In another study, BHT inhibited Fe^2+^- induced mitochondrial lipid peroxidation (23). 

The increased levels of ROS and lipid peroxidation in isolated kidney mitochondria following UA treatment showed compromised integrity of mitochondrial membrane via oxidation of membrane phospholipids. However, beta-glucan and BHT pretreatment significantly decreased UA-induced mitochondrial membrane damage (Figure 4). GSH have a known role in the mitochondrial pore because when protein thiol groups from the inner mitochondrial membrane become oxidized, the conformational changes occur in the pore complex leading to the mitochondrial permeability transition (MPT) (40). On the other hand, mitochondrial ROS can also cause cross linking and oxidation of thiol groups in the mitochondrial membrane protein which leads to MPT (41). Previous findings have demonstrated that UA induces MMP collapse and mitochondrial swelling which is a result of an increase in membrane permeability and opening of the MPT pores (4-6, 11). Conversely, MPT is considered as an initial step of apoptosis and MPT pore opening that leads to the release of apoptogenic factor such as cytochrome c from the mitochondria (41). In our study, beta-glucan and BHT reversed the UA-induced MMP collapse and mitochondrial swelling and significantly prevented UA-induced cytochrome c release. These data were in accordance with previous study which showed that BHT significantly inhibited triol-induced oxidative damage, MMP collapse and the release of cytochrome c from isolated mice liver mitochondria (22). Kakkar *et al. *also showed that BHT reversed the swelling resulted from Fe^2+^ in isolated rat liver mitochondria (21). BHT also inhibited Fe^(2+)^/citrate-induced release of cytochrome c from rat liver mitochondria (24).

The opening of MPT pores allows unrestricted proton movement across the inner membrane, leading to uncoupling of the oxidative phosphorylation (OXPHOS) and disrupts the ATP synthesis and also reverses the ATP synthase. Under such conditions, intracellular ATP concentrations are rapidly hydrolyzed and declined (42).

In the eukaryotic cells, ATP is mainly provided through mitochondrial oxidative phosphorylation. The respiratory electron-transport chain and the ATP synthase complex are involved in the mitochondrial oxidative phosphorylation machinery. In this process, electrons are transferred to O_2_ through the electron transport. Then, the energy from the reduction of O_2_ to H_2_O is utilized to produce ATP by ATP synthase (43). We found that UA decreased the ATP level and ATP/ADP ratio in isolated kidney mitochondria. Treatment with beta-glucan and BHT attenuated the UA-induced ATP deficiency. It is likely that beta-glucan and BHT block the MPT induction and maintain MMP by inhibiting the mitochondrial oxidative stress which is required for ATP synthase.

In conclusion, beta-glucan exerts their protective effects against UA-induced mitochondrial dysfunction via ameliorating the mitochondrial oxidative stress. Moreover, the beta-glucan prevented OXPHOS disruption. We showed that beta-glucan, with its free radical scavenging activities, antioxidant properties and low toxicity; seems to be a highly promising agent in protection against UA-induced nephrotoxicity.

## References

[1] Squibb KS, Leggett RW, Mc Diarmid MA (2005). Prediction of renal concentrations of depleted uranium and radiation dose in Gulf War veterans with embedded shrapnel. Health Phys.

[2] Mc Diarmid MA, Keogh JP, Hooper FJ, Mc Phaul K, Squibb K, Kane R, DiPino R, Kabat M, Kaup B, Anderson L, Hoover D, Brown L, Hamilton M, Jacobson-Kram D, Burrows B, Walsh M (2000). Health effects of depleted uranium on exposed Gulf War veterans. Environ Res.

[3] Lim IK, Lee KH, Han BD, Jang JJ, Yun TK (1987). Uranyl nitrate induced polyuric acute tubular necrosis in rats. Yonsei Med. J.

[4] Pourahmad J, Shaki F, Tanbako sazan F, Ghalandari R, Ettehadi HA, Dahaghin E (2011). Protective effects of fungal β-(1→3)-D-glucan against oxidative stress cytotoxicity induced by depleted uranium in isolated rat hepatocytes Hum. Exp. Toxicol.

[5] Pourahmad J, Ghashang M, Ettehadi HA, Ghalandari R (2006). A search for cellular and molecular mechanisms involved in depleted uranium (DU) toxicity. Environ Toxicol.

[6] Daraie B, Pourahmad J, Hamidi-Pour N, Hosseini M-J, Shaki F, Soleimani M (2012). Uranyl Acetate Induces Oxidative Stress and Mitochondrial Membrane Potential Collapse in the Human Dermal Fibroblast Primary Cells. Iranian J. Pharm. Res.

[7] Linares V, Bell´es M, Albina ML, Mayayo E, S´anchez DJ, Domingo JL (2005). Combined action of U and stress in the rat. II. Effects on male reproduction..

[8] Taulan M, Paquet F, Maubert C, Delissen O, Demaille J, Romey MC (2004). Renal toxicogenomic response to chronic uranyl nitrate insult in mice. Environ Health Persp.

[9] Briner W, Murray J (2005). Effects of short-term and long-term depleted U exposure on open-field behavior and brain lipid oxidation in rats. Neurotoxicol. Teratol.

[10] Chomyn A, Attardi G (2003). MtDNA mutations in aging and apoptosis. BiochemBioph Res Co.

[11] Thie´baultCl, Carrie`re M, Milgram S, Simon Al, Avoscan L, Gouget B (2007). Uranium Induces Apoptosis and Is Genotoxic to Normal Rat Kidney (NRK-52E) Proximal Cells. Toxicol. Sci.

[12] Zhang X-f, Ding C-l, Liu H, Liu L-h, Zhao C-q (2011). Protective effects of ion-imprinted chitooligo saccharides as uranium-specific chelating agents against the cytotoxicity of depleted uranium in human kidney cells. Toxicology.

[13] Sener G, Toklu H, Ercan F, Erkanli G (2005). rotective effect of b-glucan against oxidative organ injury in a rat model of sepsis. IntImmunopharm.

[14] Schronerová K, Babincová M, Machová E, Kogan G (2007). Carboxymethylated (1 --> 3)-beta-D-glucan protects liposomes against ultraviolet light-induced lipid peroxidation. J. Med. Food.

[15] Babincová M, Machová E, Kogan G (1999). Carboxymethylated glucan inhibits lipid peroxidation in liposomes. Z Naturforsch C.

[16] Babincová M, Bacová Z, Machová E, Kogan G (2002). Antioxidant properties of carboxymethyl glucan: comparative analysis. J. Med. Food.

[17] Ceyhan AM, Akkaya VB, lec¸olSeCG, Ceyhan BlM, zgu¨ner FO, Chen W (2012). Protective effects of b-glucan against oxidative injury induced by 2.45-GHz electromagnetic radiation in the skin tissue of rats. Dermatol. Res.

[18] Jaehrig SC, Rohn S, Kroh LW, Fleischer LG, Kurz T (2007). In-vitro potential antioxidant activity of (1-->3),(1-->6)-beta-D-glucan and protein fractions from Saccharomyces cerevisiae cell walls. J. Agric. Food Chem.

[19] Toklu HZ, Sehirli AO, Velioglu-Ogu¨nc A, Cetinel S, Sener G (2006). Acetaminophen-induced toxicity is prevented by b-D-glucan treatment in mice. Eur. J. Pharmacol.

[20] Dacre J (1961). The metabolism of 3: 5-di-tert-butyl-4-hydroxytoluene and 3: 5-di-tert-butyl-4-hydroxybenzoic acid in the rabbit. Biochem. J.

[21] Kakkar P, Mehrotra S, Viswanathan PN (1998). Influence of antioxidants on the peroxidative swelling of mitochondria in-vitro. Cell BiolToxicol.

[22] Liu H, Wang T, Huang K (2009). Cholestane-3β,5α,6β-triol-induced reactive oxygen species production promotes mitochondrial dysfunction in isolated mice liver mitochondria. Chemico-Biological Interactions.

[23] Gogvadze V, Walter PB, Ames BN (2003). The role of Fe2+-induced lipid peroxidation in the initiation of the mitochondrial permeability transition. Archives of Biochemistry and Biophysics.

[24] Boireau A, Maréchal PM, Meunier M, Dubédat P, Moussaoui S (2000). The anti-oxidant ebselen antagonizes the release of the apoptogenic factor cytochrome c induced by Fe2+/citrate in rat liver mitochondria. Neurosci.Lett.

[25] Ghazi-khansari M, Mohammadi-Bardbori A, Hosseini M-J (2006). Using Janus Green B to Study Paraquat Toxicity in Rat Liver Mitochondria Role of ACE Inhibitors (Thiol and NonthiolACEi). Annals New York Acad. Sci.

[26] Bradford MM (1976). A rapid and sensitive method for the quantitation of microgram quantities of protein utilizing the principle of protein-dye binding. Anal.Biochem.

[27] Lambowitz AM (1979). Preparation and analysis of mitochondrial ribosomes. Methods Enzymol.

[28] Jiang GCT, Tidwell K, McLaughlin BA, Cai J C, Gupta R, Milatovic D, Nass R, Aschner M (2007). Neurotoxic Potential of Depleted Uranium-Effects in Primary Cortical Neuron Cultures and in Caenorhabditiselegans. Toxicological Sciences.

[29] Gao X, Zheng CY, Yang L, Tang XC, Zhang HY (2009). Huperzine A protects isolated rat brain mitochondria against β-amyloid peptide. Free Radical. Bio. Med.

[30] Sadegh C, Schreck RP (2003). The Spectroscopic Determination of Aqueous Sulfite Using Ellman’s Reagent. MURJ.

[31] Baracca A, Sgarbi G, Solaini G, Lenaz G (2003). Rhodamine 123 as a probe of mitochondrial membrane potential: evaluation of proton flux through F0 during ATP synthesis. Biochim BiophysActa.

[32] Zhao Y, Ye L, Liu H, Xia Q, Zhang Y, Yang X, Wang K (2010). Vanadium compounds induced mitochondria permeability transition pore (PTP) opening related to oxidative stress. J. Inorg. Biochem.

[33] TafreshiNKh, Hosseinkhani S, Sadeghizadeh M, Sadeghi M, Ranjbar B, Naderi-Manesh HT (2007). The influence of insertion of a critical residue (Arg356) in structure and bioluminescence spectra of firefly luciferase. J. Biol. Chem.

[34] Zheng W, Wang SY (2001). Antioxidant activity and phenolic compounds in selected herbs. J. Agric. Food Chem.

[35] Shaki F, Pourahmad J, Hosseini M-J, Ghazi-khansari M (2012). Toxicity of depleted uranium on isolated rat kidney mitochondria. Biochim. Biophys. Acta.

[36] Pourahmad J, Hosseini MJ, Eskandari MR, Rahmani F (2012). Involvement of Four Different Intracellular Sites in Chloroacetaldehyde-Induced Oxidative Stress Cytotoxicity. Iranian J. Pharm. Res.

[37] Pourahmad J, Mortada Y, Eskandari MR, Shahraki J (2011). Involvement of LysosomalLabilisation and Lysosomal/mitochondrial Cross-Talk in Diclofenac Induced Hepatotoxicity. Iranian J. Pharm. Res.

[38] Firat C, Samdancı E, Erbatur S, Aytekin AH, Ak M, Turtay MG, Coban YK (2013). b-Glucan treatment prevents progressive burn ischaemia in the zone of stasis and improves burn healing: An experimental study in rats. Burns.

[39] Alp H, Varol S, Celik M, Altas M, Evliyaoglu O, Tokgoz O, Tanrıverdi MH, Uzar E (2012). Protective Effects of Beta Glucan and Gliclazide on Brain Tissue and Sciatic Nerve of Diabetic Rats Induced by Streptozosin. Exp. Diabetes Res.

[40] Carvalho C, CorreiaSn, Santos MS, Seic R, Oliveira CR, Moreira PI (2008). Metformin promotes isolated rat liver mitochondria impairment. Mol Cell Biochem.

[41] Kowaltowski AJ, Netto LES, Vercesi AE (1998). The Thiol-specific Antioxidant Enzyme Prevents Mitochondrial Permeability Transition (evidence for the participation of reactive oxygen species in this mechanism). J. Biol.Chem.

[42] Halestrap AP, Clarke SJ, Khaliulin I (2007). The role of mitochondria in protection of the heart by preconditioning. Biochim. Biophys. Acta.

[43] Ludovico P, Sansonetty F, Corte-Real M (2001). Assessment of mitochondrial membrane potential in yeast cell populations by flow cytometry. Microbiology.

